# Local Effects of a 1940 nm Thulium-Doped Fiber Laser and a 1470 nm Diode Laser on the Pulmonary Parenchyma: An Experimental Study in a Pig Model

**DOI:** 10.3390/ma14185457

**Published:** 2021-09-21

**Authors:** Maciej Janeczek, Zbigniew Rybak, Anna Lipińska, Jolanta Bujok, Albert Czerski, Maria Szymonowicz, Maciej Dobrzyński, Jacek Świderski, Bogusława Żywicka

**Affiliations:** 1 Department of Animal Physiology and Biostructure, Division of Anatomy, Wroclaw University of Environmental and Life Sciences, Kożuchowska 1, 51-631 Wroclaw, Poland; maciej.janeczek@upwr.edu.pl (M.J.); anna.lipinska@upwr.edu.pl (A.L.); 2 Department of Experimental Surgery and Biomaterial Research, Wroclaw Medical University, Bujwida 44, 50-368 Wroclaw, Poland; zbigniew.rybak@umed.wroc.pl (Z.R.); maria.szymonowicz@umed.wroc.pl (M.S.); boguslawa.zywicka@umed.wroc.pl (B.Ż.); 3 Department of Animal Physiology and Biostructure, Division of Animal Physiology, Wroclaw University of Environmental and Life Sciences, C.K. Norwida 31, 50-375 Wroclaw, Poland; albert.czerski@upwr.edu.pl; 4 Department of Pediatric Dentistry and Preclinical Dentistry, Wroclaw Medical University, Krakowska 26, 50-425 Wroclaw, Poland; maciej.dobrzynski@umed.wroc.pl; 5 Institute of Optoelectronics, Military University of Technology, Kaliskiego 2, 00-908 Warsaw, Poland; jacek.swiderski@wat.edu.pl

**Keywords:** diode laser (1470 nm), thulium-doped fiber laser (1940 nm), hemostasis, pig model, thermal damage zone, lung surgery

## Abstract

The lungs are a common site of metastases from malignant tumors. Their removal with a minimal but safe tissue margin is essential for the long-term survival of patients. The aim of this study was to evaluate the usefulness of a 1940 nm thulium-doped fiber laser (TDFL) and a 1470 nm diode laser (DL) in a pig model of lung surgery that involved the incision and excision of lung tissue. Histopathological analysis was performed on days 0 and 7 after surgery. Neither TDFL nor DL caused significant perioperative or postoperative bleeding. Histological analysis revealed the presence of carbonized necrotic tissue, mixed fibrin–cellular exudate in the superficial zone of thermal damage and bands of deeper thermal changes. The mean total width of thermal damage on day 0 was 499.46 ± 61.44 and 937.39 ± 109.65 µm for TDFL and DL, respectively. On day 7, cell activation and repair processes were visible. The total width of thermal damage was 2615.74 ± 487.17 µm for TDFL vs. 6500.34 ±1118.02 µm for DL. The superficial zone of thermal damage was narrower for TDFL on both days 0 and 7. The results confirm the effectiveness of both types of laser in cutting and providing hemostasis in the lungs. TDFL caused less thermal damage to the lung parenchyma than DL.

## 1. Introduction

The lungs are a common site for malignant tumor metastases [1]. In clinical practice, metastases are removed using various techniques. These range from minimally invasive video-assisted thoracic surgery (VATS) to invasive thoracotomy, during which either anatomical or non-anatomical resections are performed [2,3,4,5]. Devices that provide good hemostasis together with pneumostasis and good cutting precision with minimal loss of functional pulmonary parenchyma during surgery are desired. There are several possibilities; however, each has its own limitations. Staplers provide good hemostasis but may be associated with an inappropriate postoperative tissue margin and the removal of a larger than necessary amount of lung tissue, which is disadvantageous, especially in multiple metastases. Electrocauteries have excellent cutting properties but typically do not provide adequate hemostasis in lung tissue, which necessitates additional suturing or the use of surgical sealants [6,7]. Since the early 1990s, reports on the use of lasers in lung metastasectomy have become increasingly common. Most commonly, the neodymium-doped yttrium aluminum garnet (Nd:YAG) laser operating at a 1318 nm wavelength has been utilized both in experimental and clinical studies [8,9,10,11,12]. An increased wavelength of the emitted laser light compared to 1064 nm Nd:YAG lasers seems to provide better surgical outcomes in terms of cutting and coagulation efficacy [11,12]. Laser-assisted partial lung resection is mainly reported to be suitable in open surgery; however, case reports on laser-assisted VATS have been published recently [13,14]. Considering the parenchymal structure of the lungs, which is characterized by low electrical conductivity and good thermal isolation, larger areas of lung tissue than other soft tissue can be removed using the same energy beam [15]. Strong tissue heating leads to the formation of a necrotic zone encompassing the tumor and the margin of healthy tissue. However, the thermal energy of the laser beam can be minimized as it reaches blood vessels or airways by means of heat dissipation into surroundings [16]. Hence, the power of lasers used to remove metastases in centrally located areas of the lungs has to be adequately adjusted in order avoid serious tissue damage. Laser-assisted lung surgery, mainly with Nd:YAG lasers, has become increasingly popular. Available data suggest that there is a low risk of local recurrence following laser ablation, allowing the resection of more nodules (independent of their location) while preserving the pulmonary parenchyma and minimizing postoperative complications [17]. Lasers can be used to excise more metastases while achieving an equivalent long-term survival rate and enabling re-operations when necessary [18]. Some previous studies have even demonstrated the superiority of laser ablation compared with standard surgical procedures in terms of the success of radical resection, patient survival rates, the simplicity of video-guided laser procedures during minithoracotomy, and the possibility of intra-operative lung palpation [19,20]. In summary, laser use in lung surgery is highly promising due to the fact of its prevention of hemorrhaging during and post-surgery, its preservation of healthy lung tissue as well as the possibility it provides of removing cavernous lesions in sites that were previously inaccessible surgically [21]. In addition, small peripheral lung defects following resection using the Nd:YAG laser did not have to be sutured, as the laser-induced vaporization of the lung parenchyma detected in studies seemed tightly sealed [22,23].

Several types of laser are used for soft-tissue surgery, i.e., carbon dioxide (CO_2_) with an admixture of neodymium yttrium aluminum garnet (Nd: YAG) or diode lasers [24,25,26]. More recently, not only diode lasers (DLs) but also thulium-doped fiber lasers (TDFLs) have been introduced to operating theaters and have been utilized in various procedures, many times providing excellent surgical outcomes as evaluated by hemostatic potential, cutting efficacy and precision, and sparing of the surrounding tissue [27]. Experimental data on the use of DLs and TDFLs in soft tissue surgery are also promising [28,29,30]. Both lasers operate at higher wavelengths closer to the peak absorption of water, especially thulium lasers which emit light with a wavelength of approximately 2000 nm [31]. This could potentially increase safety, precision, and oncological radicality in metastasis removal, thus minimizing postoperative complications and improving long-term outcomes. According to our knowledge so far, reports on the use of these lasers in lung surgery are limited to ex vivo experimental studies and a few small retrospective and randomized trials [13,14,32,33,34].

The aim of our study was a comparative analysis of the effect of lung cutting and resection in a pig model using a continuous-wave Tm^3+^ full-fiber diode pumped 1940 nm laser (TDFL) and 1470 nm diode laser (DL) on the degree of pulmonary tissue damage and bleeding peri- and postoperatively.

## 2. Materials and Methods

### 2.1. Lasers

Two solid-state lasers constructed at the Military University of Technology in Warsaw in cooperation with Metrum Cryoflex Sp. z o. o. (Blizne Łaszczyńskiego, Poland) were used in this study. These included a semiconductor multimode diode laser (DL), operating at a wavelength of 1470 nm, and a high-power thulium-doped fiber laser (TDFL) emitting light at a wavelength of 1940 nm. The DL provided an output continuous-wave (CW) power of up to 50 ± 1 W at a wavelength of 1470 nm. It could operate in both CW and quasi-CW (QCW) modes of operation, being able to generate optical pulses with durations of 200 ms or more. The TDFL generated an output CW power of 21 ± 1 W but, contrary to the diode laser, in a single-mode beam, which is only diffraction-limited. Such high optical beam quality generated from a fiber with a core diameter of 25 μm or smaller (and an NA below 0.1) facilitates easy light coupling to silica-based fiber probes with a diameter smaller than 50 μm. In the case of both lasers, the optical beam was directly launched from the laser probes with a diameter of 400 μm and a numerical aperture of 0.22. Based on these parameters, the power density at the fiber probe outputs was calculated to be 39.8 kW for the 1470 nm laser and 16.7 kW for the 1940 nm laser. Furthermore, the devices were used as a scalpel knife and were pulled directly across the lung’s surface with continuous motion at a constant average speed of 3.34 ± 0.38 mm/s. During operation, the lasers’ parameters were kept constant for each application of the same laser.

### 2.2. Experimental Animals

All experimental procedures were approved by the Local Ethical Committee (Approval No. 87/2012) and complied with the EU directive (2010/63/EU). The study was carried out on 8 gilts of Large Polish White breed weighing an average of 30 kg. The pigs came from the National Research Institute of Animal Production, Experimental Station in Pawłowice, Poland. Gilts were assigned to two equal groups. Animals in the first group underwent partial lung resection with the TDFL, and those in the second group underwent partial lung resection with the DL, respectively. Animals from both groups had incisions proximally and distally to the resection made with both lasers. Gilts were humanely euthanized seven days after surgery.

### 2.3. Animal Preparation and Surgery

The study was conducted on 8 gilts. In the two-week preoperative period, the animals were subjected to daily observations and veterinary examinations. Pigs were fasted for 24 h before the surgical procedure. Animals underwent partial resection and lung incisions using TDFL and DL under general anesthesia.

To induce anesthesia, the animals were administered medetomidine and butophanol intramuscularly (0.1 mg/kg body weight, Domitor^®^, Orion Pharma, Warsaw, Poland, and 0.2 mg/kg body weight, Butomidor^®^, Richter Pharma AG, Wels, Austria, respectively). Pigs were placed in the dorsal position and a bolus of propofol was administered (4 mg/kg, Scanofol^®^, Scan Vet Sp. z o. o., Skiereszewo, Poland). After intubation, animals were placed in a right lateral recumbency and kept under anesthesia with 1.5 vol.% isoflurane and continuous infusion of fentanyl (0.003 mg/kg body weight/h), Polfa SA, Łódź, Poland). Metamizole was administered perioperatively for analgesia (30 mg/kg body weight for 3 days, Biovetalgin^®^, Biowet Drwalew, Drwalew, Poland). During the procedure, animals breathed spontaneously. The partial pressure of end-tidal carbon dioxide and peripheral blood oxygen saturation were monitored using a cardiorespiratory monitor. When needed, manual ventilation was used.

The left lung was accessed by an incision of the skin and muscles in the fifth intercostal space. A surgical wound was dilated with a Weitlaner retractor, and the lung ligament was cut. After pleural sac opening, the dorsal part of the left caudal lung lobe was exposed.

The working tip of the laser was held in the hand in the manner of a pencil. The laser beam was emitted only when the working tip was positioned directly in the place where it was intended to work.

The exposed lung was incised twice with both TDFL and DL at a distance of about 2 cm. An apical fragment of the left caudal lobe with one series of incisions was excised using TDFL in the first group and with DL in the second group, respectively. Tissues were immediately fixed in formaldehyde. The site of resection was observed for several minutes to monitor for bleeding, and then the chest cavity was closed in layers.

All animals were weaned from anesthesia without complications and placed in individual boxes. They were fed ad libitum and had free access to water. On day seven post-surgery, pigs were euthanized by an intravenous pentobarbitone injection (up to 120 mg/kg, Morbital^®^, Biowet Puławy, Puławy, Poland) and the fragments of the lungs with incisions were harvested for histopathology.

### 2.4. Post-Mortem Examination

Animal cadavers were subjected to post-mortem examination. The lungs were inspected in detail at the site of incisions and resection. Then, fragments with thermal changes were harvested for histopathology.

### 2.5. Histopathology

Lung fragments were fixed for 72 h (at room temperature) in 10% buffered formaldehyde solution which was previously neutralized with calcium carbonate. Fixed fragments of lung parenchyma were cut transversely to the laser incision and cutting line into a series of tissue blocks of approximately 1 cm^3^.

The blocks of tissue were dehydrated first in acetone at 56 °C, then in xylene at room temperature and embedded in paraffin. Thus, prepared samples were then cut into approximately 4 μm thick sections using the Leica 2025 rotary microtome (Leica Microsystems, Wetzlar, Germany). The specimens were stained with hematoxylin and eosin (HE; Sigma–Aldrich, Saint Louis, MO, USA) and then enclosed in a mounting medium (CV Mount Medium, Leica Biosystems GmbH, Nussloch, Germany).

Tissue slides were evaluated under a light microscope (Olympus BX43, Olympus Corporation, Tokyo, Japan) using the cellSens standard analysis and acquisition imaging software (Version 1.6; 2010 Olympus Corporation, Tokyo, Japan) for comparisons. At least five tissue slides were evaluated from each animal for a particular laser cut, and the average values from a series of measurements were considered in statistical analysis.

### 2.6. Statistics

Experimental data are expressed as the mean ± standard deviation of data from the animals. The mean values of the measurements performed in the five to seven slides from each swine were included in the statistical analysis. Data were analyzed using the two-sample, two-tailed Mann–Whitney test. Statistica for Windows Version 10.0 software package (StatSoft, Tulsa, OK, USA) was used for the analysis. Differences were considered significant when *p* ˂ 0.05.

## 3. Results

### 3.1. Intraoperative and Post-Mortem Examination

**Figure 1:**
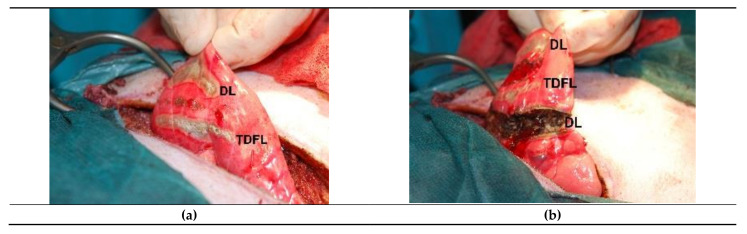
Intraoperative view of the lung: (**a**) Incisions with the diode laser (DL) and thulium-doped fiber laser (TDFL); (**b**) partial resection of the lung with the DL and incisions with both lasers above.

During incision of the lung with both TDFL and DL, no bleeding was visible. Partial lung resection with DL produced minimal bleeding, similar to TDFL (Figure 1).

**Figure 2:**
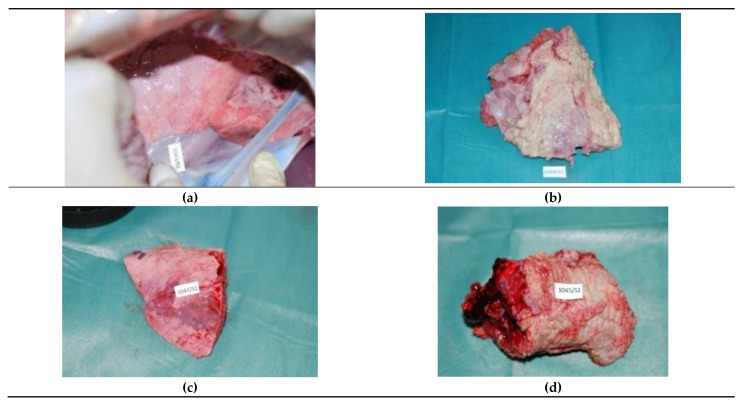
Macroscopic image of pig lungs on day seven after experimental laser surgery: (**a**,**b**) lung tissue after thulium-doped fiber laser (TDFL) partial resection; adhesion with diaphragmatic pleura is visible (**a**); (**c**,**d**) lung fragment after partial resection with diode laser (DL).

On day seven post-surgery, lungs at the site of partial resection with both the TDFL and DL formed adhesions with the diaphragmatic pleura. After the DL cutting, the adhesions were usually more extensive. Macroscopically, locally at the site of laser cuts, the tissue color was altered, and fibrosis and atelectasis were present. On the surface of the lung, incisions were poorly visible. Along the incisions, necrosis was present (Figure 2).

### 3.2. Histopathology

**Figure 3:**
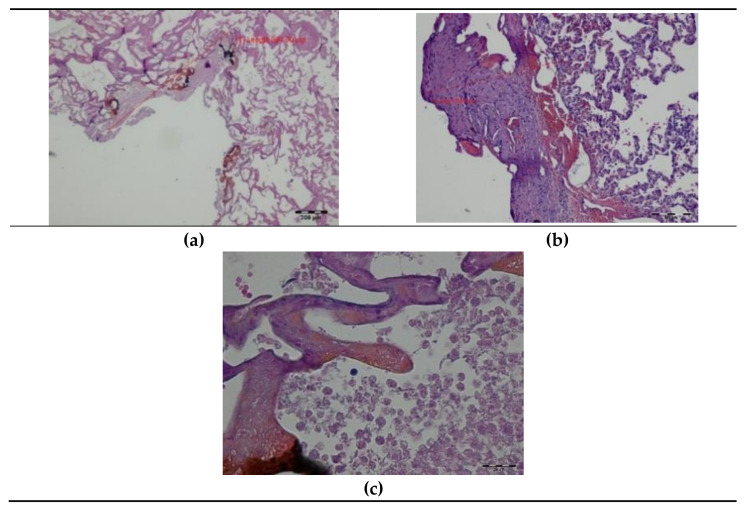
Histopathology of the pig lung tissue fragments after diode laser (DL) cutting on day 0: (**a**) total width of the thermal damage zone, magnification 40×; (**b**) superficial zone of thermal damage, magnification 100×; (**c**) superficial zone of thermal damage, magnification 400×, HE staining.

In the samples taken intraoperatively (day 0), carbonized tissue structures and an exudative phase with a small number of coagulated erythrocytes were visible at the site of the DL cut. Superficial thermal changes had an average width of 335.29 µm. Underneath, a zone of shrunken lung tissue structures was observed, with the cell structure and nuclei being preserved. The total width of visible thermal lesions was approximately 937.39 µm (Figure 3).

**Figure 4:**
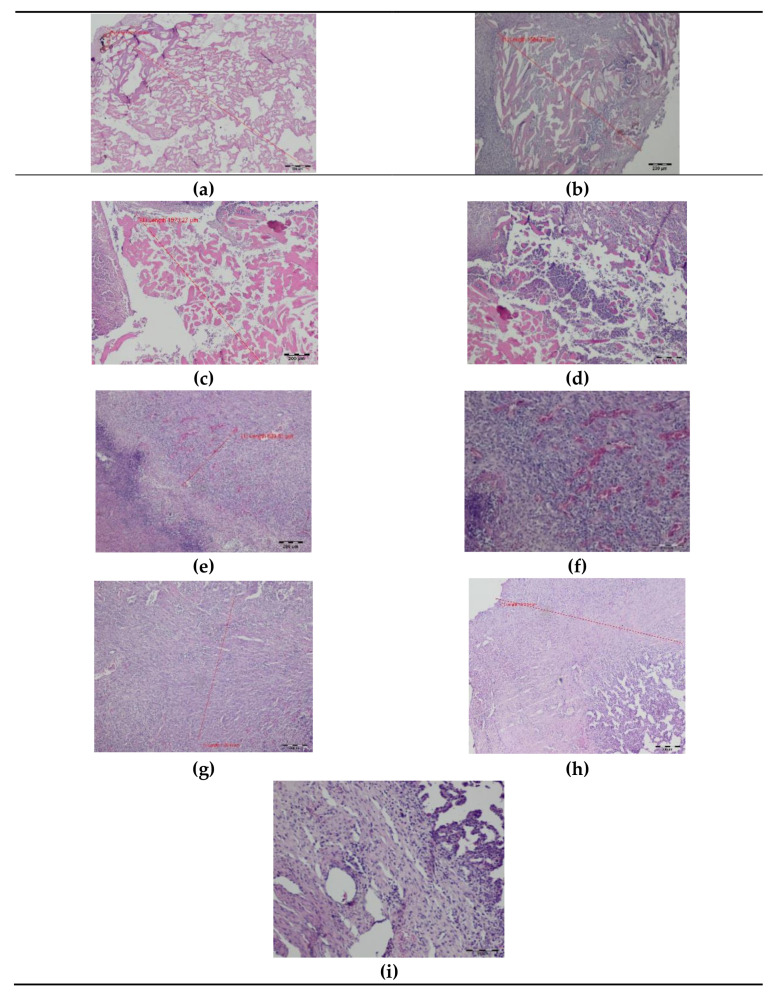
Histopathology of the pig lung seven days after surgery with a diode laser (DL): (**a**–**c**) superficial thermal changes after DL laser cutting, magnification 40×; (**d**,**e**) granulocyte infiltration zone, magnification 40×; (**e**,**f**) granulation tissue band, magnification 40× (**e**); 100× (**f**); (**g**,**h**) connective tissue proliferation (fibrosis), magnification 40×; (**i**) border between thermal damage and healthy tissue, magnification 100×; HE staining.

On day seven after surgery with DL, a zone of tissue and cellular changes were visible in the lungs. At the site of the laser cutting, remnants of the exudative phase with extravasated erythrocytes and residues of carbonized tissues were present. The average width of the superficial thermal changes was 1800.28 µm. Deeper, a zone of granulocyte infiltration with a predominance of neutrophils appeared, followed by a semicircular band of inflammatory granulation tissue with numerous thin-walled blood vessels and fibroblast proliferation quite sharply delimited from the lung tissue with a normal appearance. The total width of the laser-related thermal changes was, on average, 6500.34 µm (Figure 4). Both superficial and total zones of thermal damage were significantly wider after the seventh day compared to day 0.

**Figure 5:**
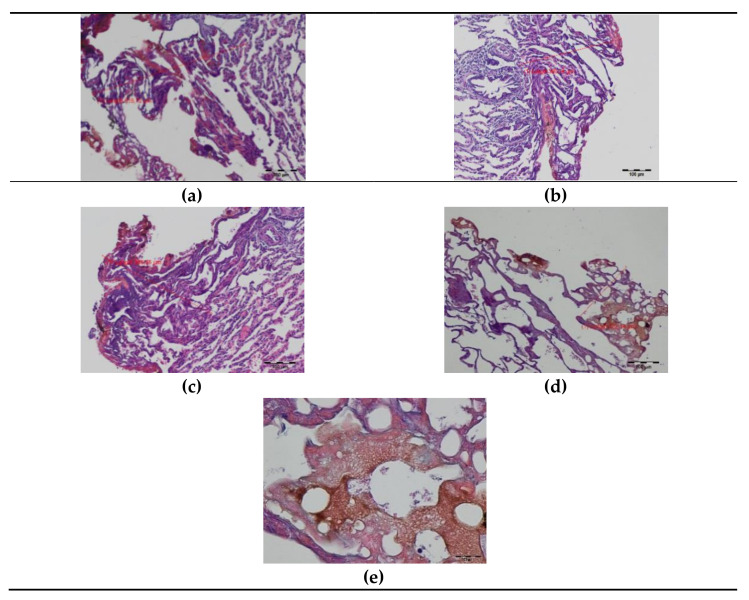
Histopathology of the lung tissue after a thulium-doped fiber laser (TDFL) cut on day 0: (**a**–**c**) total zone of laser thermal damage, magnification 100×; (**d**,**e**) superficial zone of laser thermal damage, magnification 100× and 400×; HE staining.

In the histopathology of the lung tissue taken intraoperatively (day 0), a superficial zone of carbonization and an exudative phase without extravasated erythrocytes were visible after cutting with TDFL. The superficial zone of thermal damage had an average width of 229.74 µm. Deeper, there was a band of shrunken lung tissue with a preserved cellular structure and cell nuclei. The total width of laser thermal damage was approximately 499.46 µm (Figure 5). Both the superficial and total widths of thermal damage were narrower after TDFL than after DL.

**Figure 6:**
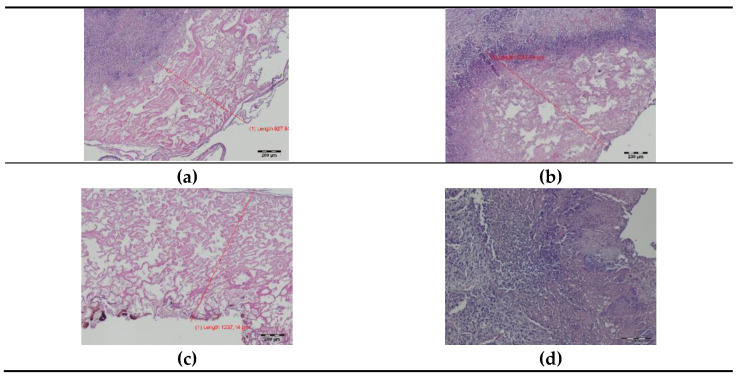

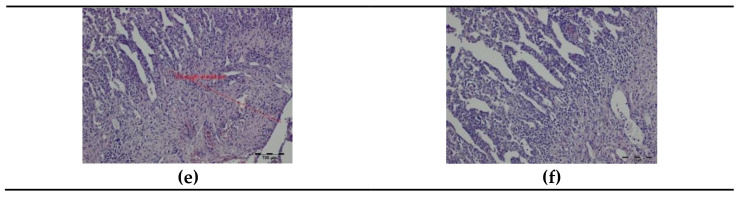
Histopathology of the lungs seven days after cutting with a thulium-doped fiber laser (TDFL): (**a**–**c**) superficial zone of thermal damage, magnification 40×; (**d**,**e**) deeper zone of granulation and connective tissue, magnification 40× (**d**); 100× (**e**); (**f**) a border between laser thermal damage zone and a healthy lung tissue, magnification 100×; HE staining.

On day seven, the remnants of the exudative phase and carbonized lung tissue were visible superficially. They were surrounded by a wide band of granulation and proliferating connective tissue with a predominance of fibroblasts, mesenchymal cells, and pneumocytes. The proliferative phase was bordered by healthy lung tissue. The average widths of the superficial and total zones of thermal damage were 1256.69 and 2615.74 µm, respectively (Figure 6). The widths of thermal changes on day seven were significantly narrower after TDFL than DL (Table 1). Similar to DL, the superficial and total zones of thermal damage were more extensive after seven days than at the time of surgery.

## 4. Discussion

Lasers are widely used in various fields and have important applications in medicine. In soft tissue surgery, the laser offers precise removal of a given area, securing an optimal margin without the need to excise healthy tissue. When passing through a tissue, the laser beam cauterizes and/or coagulates it, which significantly reduces the resulting trauma compared to classic surgery and, thus, may shorten the patient’s recovery time. The laser produces a more predictable incision than radiofrequency (RF) ablation, in that the electrical conductivity does not affect heat distribution. Lasers also produce more predictable adjacent tissue injury compared to monopolar cutters [7]. One reason for this is that the beam of light produced by the laser is delivered almost entirely in a straight line, and its side scattering is minimal. Lasers are generally well tolerated by patients, and large lesions can be removed using several laser light applicators [2].

We attempted to compare two types of lasers for lung surgery in our study: a diode laser and a thulium-doped fiber laser. The literature on this topic remains scarce. Positive reports on the clinical use of thulium lasers in prostate resection surgery, otolaryngology, and neurology as well as in experimental resection of the liver and spleen led us to perform a study assessing lasers in the parenchymal tissue of pig lungs [28,29,30,35,36,37,38,39].

TDFLs are equipped with a fiber emitting light at a wavelength of 1940 nm. Their energy absorption in water is about 1000 times greater than conventional Nd:YAG lasers emitting at a wavelength of 1064 nm. The high energy absorption assures accurate tissue cutting. The diode laser we used emits light with a wavelength of 1470 nm, which is absorbed 40 times more by water than the light emitted by Nd:YAG lasers [28,29,30,31,32]. Our main goal was to investigate whether these medium-power lasers have similar cutting precision and hemostasis comparable to high-power lasers, and whether they can be used as alternative tools in lung parenchyma surgery.

When used in lung surgery, the laser has several basic functions. The laser beam precisely cuts the encountered tissue and causes its coagulation, which leads to secondary lung tissue contraction. This contraction activates a positive feedback loop, causing the enhancement of coagulation and the sealing of the pulmonary parenchyma, which are beneficial for the resection procedure in terms of hemostasis and airtightness [22]. It was demonstrated that tissue sealing at the resection site depends on the thickness of the coagulation zone. It can be expected that the higher the laser power and the thicker the fiber of the laser, the wider the coagulation zone [21,23,32]. Such sealing reduces the likelihood of fistula formation or post-resection air leakage and respiratory unsealing, which are commonly seen after resections with electrocautery or surgical stapling. According to many authors, there is no need to secure the lung parenchyma with clips or other sealants in the case of removing single small lesions with a laser [40]. Moreover, laser-assisted surgery seems to be a safe and effective alternative to lobectomy in cases of large or central metastases where the SWR is technically impossible and mechanical resection can cause deformation and distortion of the lung parenchyma [17,18,19,41,42,43]. Studies have found that the Nd:YAG laser (1318 nm) is a tool that cuts, coagulates, seals, and stops bleeding as well as sterilizing wound edges in an area of up to 5 mm, which may improve the oncological radicality of the resection [17,44,45]. This technique has been positively assessed in terms of its facility, the low percentage of perioperative complications, completeness of resection, and oncological results [19,41,44,46]. In the retrospective study by Stefani et al. (2019), laser-assisted resection of large and centrally located lung masses resulted in a better immediate surgical outcomes and shorter hospital stay, while the recurrence rate and survival times did not differ when compared with lobectomy [40].

We successfully incised and partially excised lung tissue in a pig model using TDFL and DL without intraoperative bleeding. During the autopsy, 7 days after surgery, we observed normal wound healing after the use of both lasers. There were no signs of bleeding or blood clots in the thoracic cavity. Previously, the Nd:YAG laser was reported to provide good hemostasis in a porcine model compared to a monopolar cutter [7]. In another study, electrocautery was associated with blood loss during a partial lung resection procedure as well [47]. Stapler resection helps to overcome the hemostasis problem; however, when compared to laser resection, it is associated with a greater loss of remaining functional lung parenchyma, especially in cases of larger or multiple masses [40]. There are only a few reports on the use of TDFL or diode lasers for lung surgery where hemostasis could be evaluated in vivo. Both prevented blood loss during procedures including segmentectomy or lobectomy (TDFL) and pulmonary nodule excision (TDFL, DL) [16,17,37]. In a study by Scanagatta et al. on tissue specimens collected during lung nodule resection, a 2000 nm thulium laser was postulated to have less hemostatic power compared to a 1318 nm Nd:YAG laser; however, it provided better tissue preservation, allowing recognition of larger vessels before cutting them [33,34]. In our previous studies, both lasers provided good hemostasis when used in different soft tissues [28,29,30].

Both tested lasers precisely cut the lung parenchyma and left a narrow surrounding tissue margin. Macroscopically, the band of the necrotic lung lesions following the DL incision was wider than following a TDFL incision. DL also produced a wider zone of coagulation. When examined under a light microscope, the total area of thermal changes was much larger following the use of the diode laser (937.39 ± 109.65 µm on day 0; 6500.34 ± 1118.02 µm on day 7) than following the application of TDFL (499.46 ± 61.44 µm on day 0; 2615.74 ± 487.17 µm on day 7). Similar results were obtained following laser resection of liver, spleen, and kidney soft parenchyma [28,29,30].

To date, the data on the use of these laser types in the resection of lung tissue are sparse. Previous metric studies on different types of devices have focused on the degree of precision and cutting depth of tissue based on the type of laser used, laser settings, and operation mode [12,21,23,48]. Comparison of two Nd:YAG lasers (1064 nm; 1320 nm) showed that increased wavelength produces a sealing effect, decreasing the risk of airtightness loss, which was found to be the effect of a more favorable vaporization-to-coagulation ratio [12]. It is believed that coagulation of the lung parenchyma leads to the collapse and contraction of the alveoli and the formation of a sealing layer that helps to prevent air leakage and secondary edema and fistula formation. In experimental studies, a ND:YAG laser immediately produced a coagulation zone of approximately 1500–2000 µm, and the effect depended on the output power and cutting speed [12,23,48]. In our study, both lasers produced a total area of thermal damage of less than 1000 µm. A 2010 nm thulium laser operated at 60 W caused thermal damage of approximately 595 µm in human lung parenchyma, which is comparable to our study. A narrow zone of thermal damage provides better cutting precision; however, it might be accompanied by a weaker sealing effect and subsequent air leaks. When checked in an ex vivo model, TDFL provided initial airtightness in up to 2 cm deep resections with a mean bursting pressure of 33.7 ±  4.8 mbar when a fiber with a 600 µm diameter was used [32]. In a clinical study on anatomic pulmonary resections with incomplete fissures performed with a thulium laser emitting light at a wavelength of 2010 nm, the immediate sealing effect was satisfactory; however, in three patients, delayed air leakage occurred, and there was a necessity for additional tube placement or reoperation [33,34]. We did not observe a delayed pneumothorax in pigs on day 7 after surgery with both TDFL and DL, which is promising for healthy tissue sparing during partial lung resections. However, further studies on the feasibility of the thulium-doped fiber laser in lung surgery, emphasizing the evaluation of the durability of the surgical closure, ideally with burst pressure measurement, are advisable prior to introducing this laser into clinical use. There are no data on the sealing properties of the 1470 nm diode laser in lung parenchyma but considering the wider thermal damage zone produced by this laser type as compared to TDFL, it might be superior in partial lung resections in terms of airtightness.

Laser-assisted partial lung resections typically produced better immediate results with fewer complications and a shorter hospital stay and enabled the sparing of more functional lung parenchyma. Long-term outcomes, such as survival and local recurrence, were not inferior to the classical approach [40]. This prompts further research on the use of these lasers, especially in the case of multiple metastatic lesions in the lungs, where obtaining a narrow band of thermal lesions, but ensuring lung tightness at the same time, would leave much more functional lung tissue than with a stapler.

## 5. Conclusions

Both examined lasers produced a narrow zone of thermal damage in lung parenchyma, with TDLF producing less superficial and deeper changes while providing good hemostasis. Lasers seem to be promising for clinical application; however, additional studies are necessary to confirm this finding.

## Figures and Tables

**Table 1:** Laser thermal damage zone in pig lung tissue on days 0 and 7 after surgery with the diode laser and thulium-doped fiber laser (µm).

Time	*n*	Thulium-Doped Fiber Laser (TDFL)	Diode Laser (DL)
Day 0			
Superficial zone of thermal damage	5	229.74 ± 34.59 ^a^	335.29 ± 16.07 ^b^
Total zone of thermal damage	5	499.46 ± 61.44 ^a^	937.39 ± 109.65 ^b^
Day 7			
Superficial zone of thermal damage	5	1256.69 ± 186.44 ^a^	1800.28 ± 206.41 ^b^
Total zone of thermal damage	5	2615.74 ± 487.17 ^a^	6500.34 ± 1118.02 ^b^

Values expressed as the mean ± standard deviation; *n*—number of animals; ^a,b^—different letters in superscript in a row indicate significantly different values.

## Data Availability

The data presented in this study are available on request from the corresponding author.
